# Arthroprotective Effects of Cf-02 Sharing Structural Similarity with Quercetin

**DOI:** 10.3390/ijms19051453

**Published:** 2018-05-14

**Authors:** Feng-Cheng Liu, Jeng-Wei Lu, Chiao-Yun Chien, Hsu-Shan Huang, Chia-Chung Lee, Shiu-Bii Lien, Leou-Chyr Lin, Liv Weichien Chen, Yi-Jung Ho, Min-Chung Shen, Ling-Jun Ho, Jenn-Haung Lai

**Affiliations:** 1 Rheumatology/Immunology and Allergy, Department of Medicine, Tri-Service General Hospital, National Defense Medical Center, No. 161, Section 6, Minquan East Road, Taipei 114, Taiwan; lfc10399@yahoo.com.tw (F.-C.L.); epichien@gmail.com (C.-Y.C.); mslivcat@gmail.com (L.W.C.); 2 Department of Biological Sciences, National University of Singapore, 14 Science Drive 4, Singapore 117543, Singapore; jengweilu@gmail.com; 3 Graduate Institute of Cancer Biology and Drug Discovery, College of Medical Science and Technology, Taipei Medical University, Taipei 110, Taiwan; huanghs99@tmu.edu.tw (H.-S.H.); levi0963309363@gmail.com (C.-C.L.); 4 Department of Orthopaedics, Tri-Service General Hospital, National Defense Medical Center, No. 161, Section 6, Minquan East Road, Taipei 114, Taiwan; LSB3612@yahoo.com.tw (S.-B.L.); lchlin66@hotmail.com (L.-C.L.); 5 School of Pharmacy, National Defense Medical Center, No. 161, Section 6, Minquan East Road, Taipei 114, Taiwan; ejung330@gmail.com; 6 Rheumatology/Immunology and Allergy, Department of Medicine, Armed Forces Taoyuan General Hospital; Taoyuan 325, Taiwan; airfly100@gmail.com; 7 Institute of Cellular and System Medicine, National Health Research Institute, No. 35, Keyan Road, Zhunan, Miaoli County 350, Taiwan; lingjunho@nhri.org.tw; 8 Graduate Institute of Microbiology and Immunology, National Defense Medical Center, No. 161, Section 6, Minquan East Road, Taipei 114, Taiwan; 9 Division of Allergy, Immunology and Rheumatology, Department of Internal Medicine, Chang Gung Memorial Hospital, Chang Gung University, No. 5, Fusing St., Gueishan Township, Tao-Yuan County 333, Taiwan

**Keywords:** arthritis, osteoarthritis, rheumatoid arthritis, small-molecule inhibitor, chondrocytes, tumor necrosis factor-alpha, inflammation

## Abstract

In this study, we synthesized hundreds of analogues based on the structure of small-molecule inhibitors (SMIs) that were previously identified in our laboratory with the aim of identifying potent yet safe compounds for arthritis therapeutics. One of the analogues was shown to share structural similarity with quercetin, a potent anti-inflammatory flavonoid present in many different fruits and vegetables. We investigated the immunomodulatory effects of this compound, namely 6-(2,4-difluorophenyl)-3-(3-(trifluoromethyl)phenyl)-2*H*-benzo[*e*][1,3]oxazine-2,4(3*H*)-dione (Cf-02), in a side-by-side comparison with quercetin. Chondrocytes were isolated from pig joints or the joints of patients with osteoarthritis that had undergone total knee replacement surgery. Several measures were used to assess the immunomodulatory potency of these compounds in tumor necrosis factor (TNF-α)-stimulated chondrocytes. Characterization included the protein and mRNA levels of molecules associated with arthritis pathogenesis as well as the inducible nitric oxide synthase (iNOS)–nitric oxide (NO) system and matrix metalloproteinases (MMPs) in cultured chondrocytes and proteoglycan, and aggrecan degradation in cartilage explants. We also examined the activation of several important transcription factors, including nuclear factor-kappaB (NF-κB), interferon regulatory factor-1 (IRF-1), signal transducer and activator of transcription-3 (STAT-3), and activator protein-1 (AP-1). Our overall results indicate that the immunomodulatory potency of Cf-02 is fifty-fold more efficient than that of quercetin without any indication of cytotoxicity. When tested in vivo using the induced edema method, Cf-02 was shown to suppress inflammation and cartilage damage. The proposed method shows considerable promise for the identification of candidate disease-modifying immunomodulatory drugs and leads compounds for arthritis therapeutics.

## 1. Introduction

Arthritis is an inflammation of the joints, the most common types are rheumatoid arthritis (RA) and osteoarthritis [1,2]. Many factors such as aging, obesity, trauma, genetic predisposition, and endocrine makeup can contribute to the development of osteoarthritis [3,4,5,6]. Several catabolic factors are known to contribute to joint damage in osteoarthritis. These include molecules such as proinflammatory cytokines (e.g., interleukin-1 (IL-1) and tumor necrosis factor-alpha (TNF-α), matrix metalloproteinases (MMPs), and aggrecanases (a disintegrin and metalloproteinase with thrombospondin motifs (ADAMTS), as well as the inducible nitric oxide synthase (iNOS)–nitric oxide (NO) system [7,8,9,10,11]. Importantly, these factors interact closely with one another. For example, the production of MMP-13 and NO can be efficiently induced by both TNF-α and IL-1 in chondrocytes [12].

Extensive genetic analysis has led to the identification of several transcription factors which act as determinants that regulate the expression of many osteoarthritis pathogenesis-contributing factors [13,14]. A series of reports from our lab, as well as work completed by other research teams, have shown that these transcription factors include nuclear factor-kappaB (NF-κB), interferon regulatory factor-1 (IRF-1), the signal transducer and activator of transcription-3 (STAT-3), and activator protein-1 (AP-1) [15,16,17]. The activities of these transcription factors faithfully reflect the status of joint inflammation and are highly predictive of joint damage in a variety of arthritis models [18]. Recent research has highlighted epigenetic factors as contributing to osteoarthritis [19].

Candidate disease-modifying antiarthritis drugs should preserve immunomodulatory effects and have very limited or no toxicity [20,21,22]. Small molecules that target specific signaling pathways and/or mechanisms have considerable potential to meet these criteria [23,24]. Screening a mini-library containing three-hundred benzamide-linked small molecules allowed us to identify three compounds that could potentially act as disease-modifying antiarthritis drugs. The three compounds are 2-hydroxy-*N*-[3-(trifluoromethyl)phenyl]benzamide (HS-Cf) [15], *N*-(4-chloro-2-fluorophenyl)-2-hydroxybenzamide (HS-Cm) [25], and *N*-(3-chloro-4-fluorophenyl)-2-hydroxybenzamide (HS-Ck). While seeking to synthesize potent derivatives from the synthesized analogues of HS-Cf, we accidentally found a novel compound, 6-(2,4-difluorophenyl)-3-(3-(trifluoromethyl)phenyl)-2*H*-benzo[*e*][1,3]oxazine-2,4(3*H*)-dione (Cf-02), which shares many structural similarities with quercetin, a potent immunomodulatory compound that is present in many different fruits and vegetables [26]. In the present study, we characterized the immunomodulatory potencies of the new compound using a variety of methods and conducted a direct comparison with quercetin. Our results revealed that the immunomodulatory potency of Cf-02 is more than fifty-fold stronger than that of quercetin, making it a strong candidate for disease-modifying drugs against arthritis.

## 2. Results

### 2.1. Inhibiting iNOS–NO Production in TNF-α-Stimulated Porcine Chondrocytes via Cf-02

**Figure 1:**
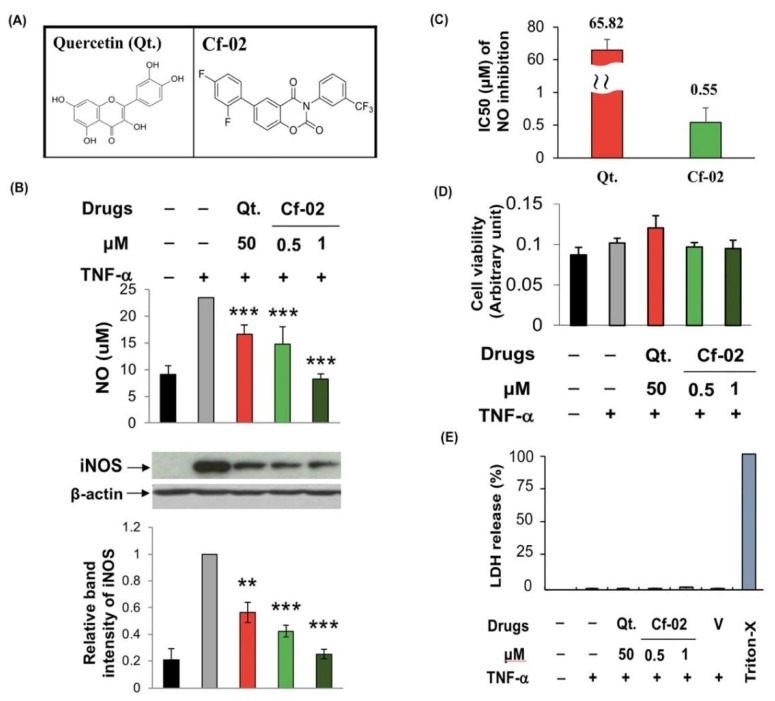
iNOS–NO production inhibited by Cf-02 in a dose-dependent manner in porcine chondrocytes stimulated by TNF-α. Structures of quercetin and Cf-02 (**A**); Porcine chondrocytes were pretreated with various doses of quercetin, Cf-02, or dimethyl sulfoxide (DMSO) for 2 h and then stimulated with TNF-α for 24 h. The expression of iNOS was determined by Western blotting according to the measurement of band intensities. The concentration of NO in the supernatant was determined using the Griess reaction (**B**); The IC_50_ for quercetin and Cf-02 were measured and given (**C**); Possible cytotoxic effects of Cf-02 were detected by treating porcine chondrocytes with Cf-02 at various concentrations for 48 h. Subsequently, the cell viability was analyzed by MTT assay. (**D**) and LDH release assays (**E**). Positive control: Equal numbers of cells treated with 1% Triton X-100 were used as the positive control. Representative data from no fewer than three independent experiments are presented in the figure. Data are mean ± SD from three independent experiments. ** *p* < 0.01; *** *p* < 0.001 compared to chondrocytes stimulated by TNF-α in the absence of Cf-02 treatment. V: vehicle (DMSO).

We performed experiments to compare the effectiveness of Cf-02 and quercetin (Figure 1A) in suppressing the activation of the iNOS–NO pathway in TNF-α-stimulated chondrocytes. At a concentration of 1 μM, both Cf-02 and quercetin significantly suppressed the production of NO and expression of iNOS in stimulated chondrocyte cells (Figure 1B). The IC_50_ value of Cf-02 was 0.55 μM (Figure 1C). Based on the (4,5-dimethylthiazol-2-yl)-2,5-diphenyltetrazolium bromide) (MTT) assay and lactate dehydrogenase (LDH) releasing assay, neither Cf-02 nor quercetin gave any detectable indication of cytotoxicity in porcine chondrocytes (Figure 1D,E).

### 2.2. Inhibiting the Production of Chondro-Destructive Enzymes via Cf-02

**Figure 2:**
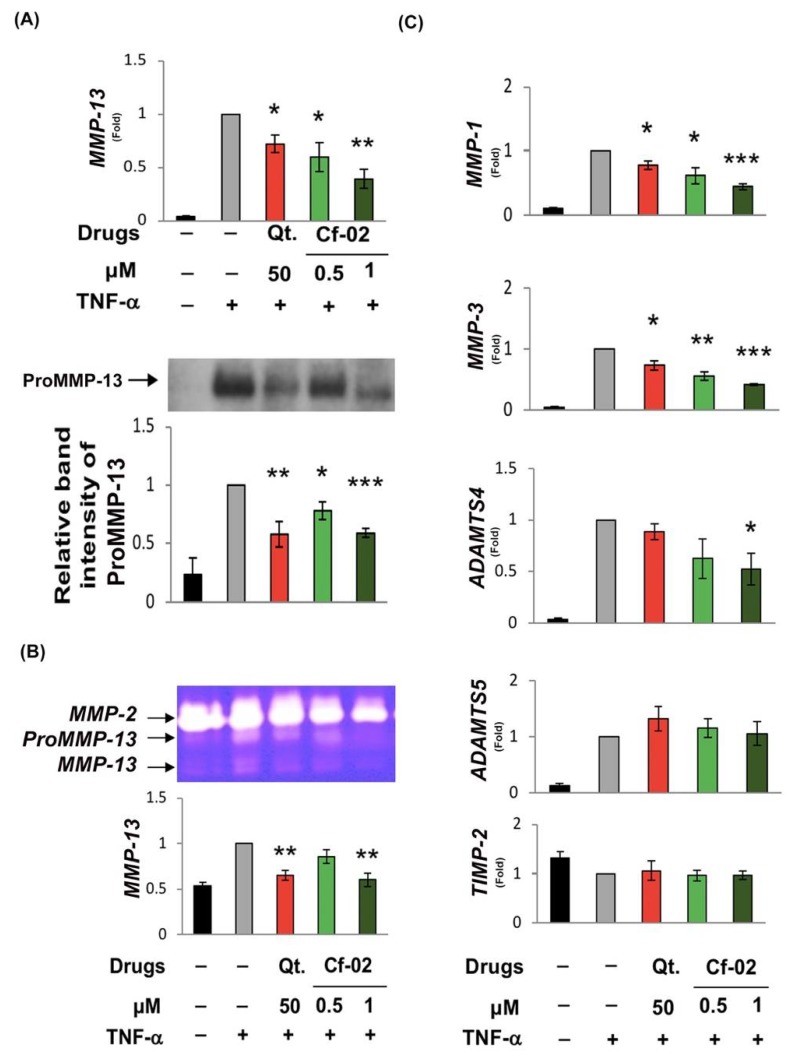
Cf-02 suppressed enzyme activity as well as the expression of TNF-α-induced matrix metalloproteinases (MMPs) and disintegrin and metalloproteinase with thrombospondin motifs (*ADAMTS*) genes. Chondrocytes were pretreated with quercetin, solvent, or Cf-02 (in various doses) for 2 h and then stimulated using 5 ng/mL TNF-α for another 8 or 24 h. Following treatment with TNF-α for 8 h, real-time RT-PCR was performed to measure the levels of *MMP-13* mRNA (**A**); Conversely, the activity of MMP-13 released into the culture supernatant was characterized using gelatin zymography following TNF-α stimulation for 24 h. Representative data pooled from at least three independent experiments are presented (**B**). Porcine chondrocytes that were treated for 2 h with various doses of quercetin, solvent, or various doses of Cf-02 were stimulated using TNF-α for 8 h. The cells were then collected for the preparation of total RNA in order to determine mRNA expression using real-time RT-PCR. The relative expression levels of *MMP-1*, *MMP-3*, *ADAMTS4*, *ADAMTS5*, and *TIMP-2* mRNA were normalized to glyceraldehyde 3-phopshate dehydrogenase (*GAPDH*), with subsequent normalization to the TNF-α-stimulated sample in each experiment (**C**). The significance of differences between sample groups was determined using one-way analysis of variance (ANOVA) with the Bonferroni *post-hoc* test. Results from three independent experiments are shown. Data are mean ± SD from three independent experiments. * *p* < 0.05; ** *p* < 0.01; *** *p* < 0.001 compared to TNF-α-stimulated chondrocytes that did not undergo Cf-02 treatment. V: vehicle (DMSO).

MMP-13 was directly responsible for damage to the cartilage matrix; therefore, we examined the effects of Cf-02 on TNF-induced *MMP-13* mRNA and MMP-13 protein expression. The results of real-time reverse transcription polymerase chain reaction (RT-PCR) and Western blot analysis revealed that (1) TNF-induced *MMP-13* mRNA expression and (2) proMMP-13 protein levels were significantly suppressed by 1 μM of Cf-02 (Figure 2A). The zymographic analysis further revealed that a Cf-02 concentration of 1 μM significantly suppressed TNF-induced MMP-13 enzyme activity (Figure 2B). Other proteinases genes, such as *MMP-1*, *MMP-3*, and *ADAMTS4* were also inhibited by Cf-02, although the intensity of the effects varied (Figure 2C). However, treatment with Cf-02 did not appear to have any effect on *ADAMTS5* and *TIMP-2* mRNA expression.

### 2.3. Regulating the Activity of Transcriptional Factors via Cf-02

**Figure 3:**
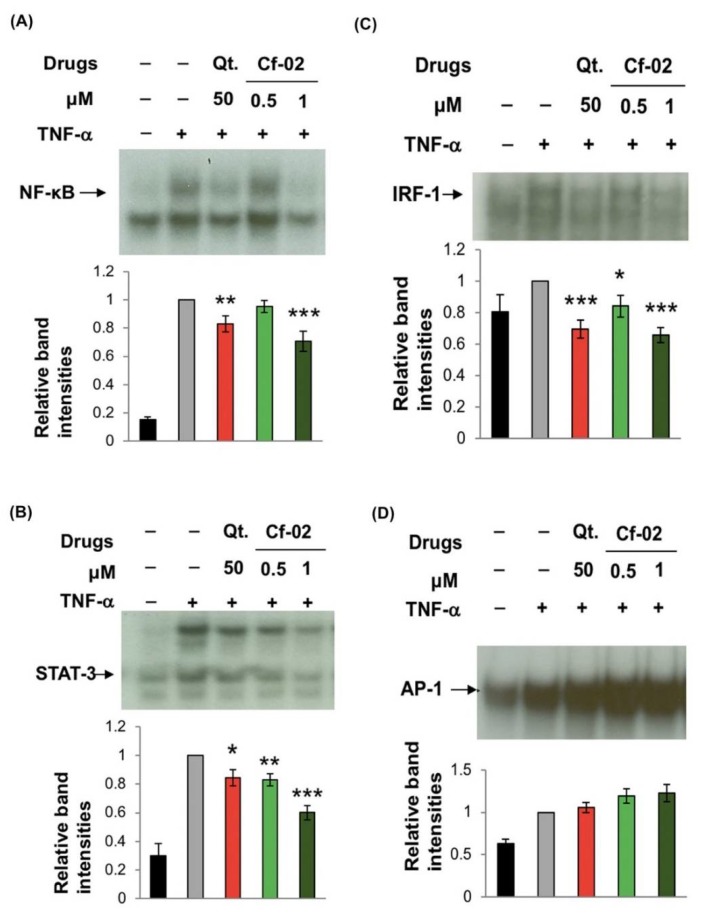
Cf-02 suppressed TNF-α induced DNA-binding of NF-κB, STAT-3, and IRF-1 but not AP-1. Nuclear extracts of chondrocytes treated for 2 h with 5 ng/mL TNF-α in the presence of quercetin, solvent, or various doses of Cf-02 were analyzed in order to quantify the DNA-binding activity of NF-κB (**A**), STAT-3 (**B**), IRF-1 (**C**), and AP-1 (**D**) with electrophoretic mobility shift assay (EMSA). For this, band intensity results were averaged from at least 3 independent experiments. Data are mean ± SD from three independent experiments. * *p* < 0.05; ** *p* < 0.01; *** *p* < 0.001 compared to TNF-α-stimulated chondrocytes that did not undergo Cf-02 treatment.

We also compared the effects of quercetin and Cf-02 on several transcriptional factors which are important to the activation of pro-inflammatory mediators in TNF-α-activated chondrocytes. Chondrocytes are stimulated by TNF-α to trigger NF-κB in the nucleus to drive downstream gene expression, thereby indicating that TNF-α-induced NF-κB DNA-binding activity was suppressed by Cf-02 (Figure 3A). Our results also showed that Cf-02 can significantly inhibit TNF-α-induced STAT-3 and IRF-1 activation (Figure 3B,C). However, Cf-02 was ineffectual in inhibiting TNF-α-induced AP-1 DNA-binding activity in chondrocytes (Figure 3D).

### 2.4. Effects of Cf-02 on TNF-α-Induced Proteoglycan/Aggrecan Degradation in Cartilage Explants

**Figure 4:**
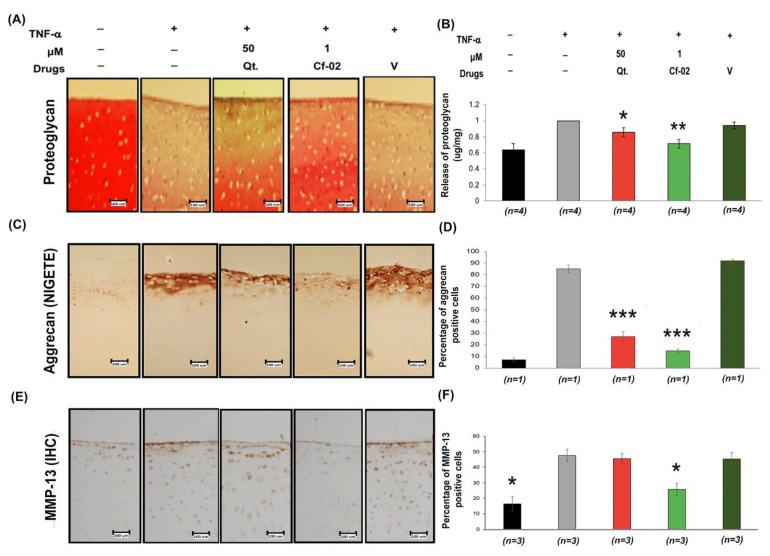
Effects of Cf-02 on TNF-α-induced proteoglycan/aggrecan degradation. In 24-well plates, the porcine cartilage blocks were cultured for 2 h with or without pretreatment with 1 μM Cf-02. Cartilage was then stimulated with 5 ng/mL TNF-α for another 72 h incubation. The proteoglycan retained in cartilage explants was monitored using Safranin-O staining (**A**) (100×). The release of proteoglycan into the culture medium was normalized with the weight of the cartilage (**B**). The intensity of aggrecan staining was examined in parallel (**C**,**D**) (100×). IHC staining of MMP-13 protein expression in porcine cartilage tissue blocks (**E**,**F**) (100×). Representative data from 3 independent experiments using cartilage from different donor blocks are presented. Data are mean ± SD from in each group. * *p* < 0.05; ** *p* < 0.01; *** *p* < 0.001 compared to TNF-α-stimulated chondrocytes that did not undergo Cf-02 treatment. Scale bars = 100 um.

We also examined the chondroprotective effects of Cf-02 in order to elucidate its anti-inflammatory properties. Specifically, our objective was to determine whether Cf-02 could be used to prevent TNF-α-induced degradation of the cartilage matrix. After treating samples with TNF-α, we observed a significant reduction in Safranin-O positive proteoglycan and an increase in the cleavage products of aggrecan (NITEGE). However, these TNF-α-induced effects were prevented by pre-treatment with Cf-02 (Figure 4). Our results consistently demonstrated the effectiveness of Cf-02 in preventing the TNF-α-mediated release of proteoglycan and aggrecan into the culture supernatants of cartilage explants (Figure 4A–D). Treatment with Cf-02 was also shown to reduce the immunohistochemistry (IHC) staining associated with MMP-13 protein expression in porcine cartilage tissue blocks (Figure 4E,F).

### 2.5. Immunomodulatory Effects of Cf-02 on Human Chondrocytes

**Figure 5:**
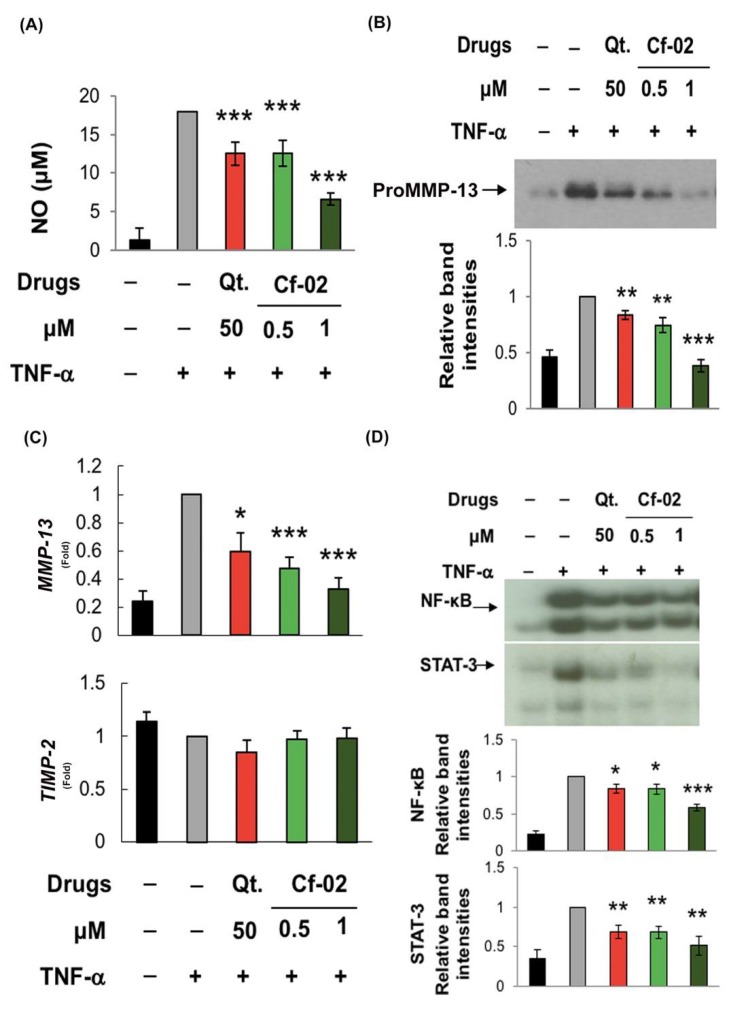
Effects of Cf-02 on TNF-α-stimulated human chondrocytes. Human chondrocytes (prepared from cartilage samples collected from patients who underwent total knee replacement) were first pretreated with quercetin, solvent, or Cf-02 in various doses for 2 h and then treated with 5 ng/mL TNF-α for an additional 24 h. Measurement of NO production (**A**), proMMP-13 expression (**B**), *MMP-13 mRNA* expression (**C**), and NF-κB and STAT-3 DNA-binding (**D**) were performed according to the same methods as those used for porcine chondrocytes. Band intensity results were averaged from at least 3 independent experiments. Data are mean ± SD from three independent experiments. * *p* < 0.05; ** *p* < 0.01; *** *p* < 0.001 compared to TNF-α-stimulated chondrocytes that did not undergo Cf-02 treatment.

To enhance the clinical significance of Cf-02, we examined human chondrocytes prepared from surgical specimens of patients with osteoarthritis patients under conditions similar to those associated with porcine chondrocytes. Unlike the results from porcine chondrocytes, the results from human chondrocytes indicated that Cf-02 and quercetin significantly inhibited the production of TNF-α-induced NO (Figure 5A). Moreover, Cf-02 and quercetin also inhibited proMMP-13 production, especially at a concentration of 1 μM of Cf-02 (Figure 5B). Cf-02 also significantly suppressed the expression of *MMP-13* mRNA in TNF-α-activated human chondrocytes (Figure 5C). However, the expression of *TIMP-2* mRNA was unaffected by the tested Cf-02. Molecular approaches further demonstrated that Cf-02 can inhibit the TNF-α-induced DNA-binding activity of NF-κB (Figure 5D). Despite variations among the assays, the Cf-02 that was the focus of this study preserved immunomodulatory effects with a potency that was approximately 50-fold efficient than that of quercetin.

### 2.6. Prevention of Collagen Loss by Cf-02 in an Arthritis Animal Model

**Figure 6:**
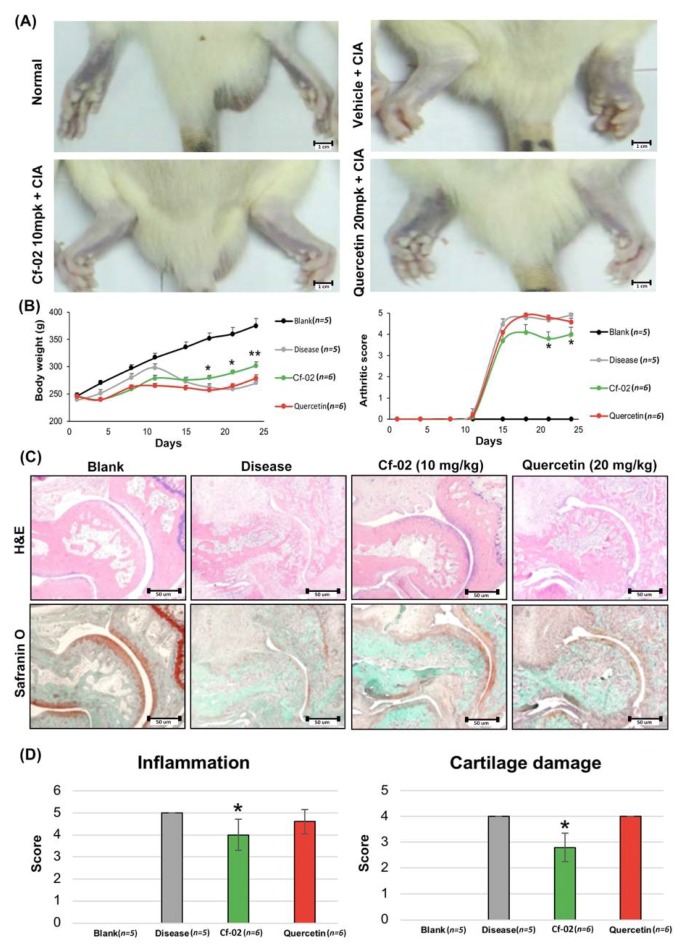
Cf-02 prevented collagen loss in an arthritis animal model. CIA rats were randomly divided into groups according to global assessments. The onset of arthritis occurred close to day 14 post first injection. Representative images of swelling joints (**A**) (25×). Body weight and arthritic scores were determined every 3 days (**B**). Representative joint sections from each group of rats at 24 days post-treatment. Hematoxylin and eosin (H&E) staining showing signs of inflammation. Safranin-O staining showing cartilage erosion (**C**) (200×). Frequency distribution of inflammation and cartilage damage scores from H&E staining results (**D**). Data are mean ± SD from in each group. The level of statistical significance was set at * *p* < 0.05. Scale bars = 1 cm (**A**), 50 um (**C**).

Cf-02 was shown to inhibit TNF-α-induced signaling, prevent the degradation of cartilage matrix, and inhibit inflammation. The anti-inflammatory activity Cf-02 was tested in vivo using a collagen II-induced edema method. As shown in Figure 6A, collagen-induced arthritis (CIA) rat treated with vehicle developed arthritis at the end of week 2, the severity of which increased throughout the study. However, in the Cf-02-treated CIA (Cf-02 + CIA) rat, the clinical manifestations of this effect were markedly inhibited. In our rat collagen-induced arthritis model, Cf-02 administered at a dose of 10 mg/kg/day was also shown to inhibit an increase in arthritis score (Figure 6B). Finally, hematoxylin and eosin stain (H&E) and Safranin-O staining (Figure 6C) indicated that Cf-02 suppressed inflammation and cartilage damage (Figure 6D).

## 3. Discussion

Previous screening of benzamide-linked small molecules in our laboratory led to the identification of three compounds with efficient anti-inflammatory activities. Among them, 2-hydroxy-*N*-[3-(trifluoromethyl)phenyl]benzamide (HS-Cf) and *N*-(4-chloro-2-fluorophenyl)-2-hydroxybenzamide (HS-Cm) had previously been reported [15,25]. Structure-based drug design was subsequently used to synthesize additional SMIs with greater potency and lower toxicity as candidates for arthritis therapeutics. Structural modifications included the introduction of a benzyl alcohol group and a fluorine substitution. Optimization of drug-like properties led to the identification of hundreds of synthesized compounds, one of which (Cf-02) shares similarities with the anti-inflammatory flavonoid quercetin. In a side-by-side comparison, Cf-02 proved more than 50 times more effective than quercetin in suppressing (1) TNF-α-induced iNOS–NO production, (2) the mRNA expression of several *ADAMTS* and *MMPs*, and (3) the enzyme activity of MMP-13 in chondrocytes. Cf-02 was also found to be 50 times more effective than quercetin in preventing the release of proteoglycan/aggrecan in cartilage explants. Molecular examinations further revealed the potency of Cf-02 in suppressing the activation of several transcription factors, including NF-κB, STAT-3, and IRF-1, but not AP-1. Our results provide evidence that Cf-02 possess chondroprotective effects and help to elucidate the mechanisms which underlie them. We also demonstrated the potential of Cf-02 to benefit the treatment of TNF-α-induced damage to the cartilage in joints. We were also to fund that as a critical transcription factor in regulating many proinflammatory genes, the TNF-α-induced DNA-binding activity of AP-1 appeared to be resistant to all compounds examined in this study. Variations between results obtained from human chondrocyte samples and results obtained from porcine chondrocytes indicate that Cf-02 possesses a certain specificity in the targeting of signaling molecules associated with inflammatory responses in arthritis.

In terms of inflammation reduction, the potency of Cf-02 exceeded that of quercetin by 50 times. In this Cf-02, the amide motif of NH and OH were cyclized to mimic the heterocyclic pyran or pyrone ring of flavonoids, which could be used to adjust its anti-inflammatory potency. Given the success in elucidating the structure–activity relationships through several different molecular and cellular bioassays, the mechanisms observed might not fully account for the subtle different bioactivities of Cf-02. The microenvironment of arthritis is very complex. Both *ADAMTS4* and *ADAMTS5* are responsible for aggrecan degradation in a human model of arthritis. However, Cf-02 only inhibits *ADAMTS4* but does not inhibit *ADAMTS5* in porcine chondrocytes [27]. Cf-02 inhibits *MMP-1*, *MMP-3*, and *MMP-13* via signal transduction by inhibiting NF-κB, STAT-3, and IRF-1, but Cf-02 was not able to inhibit MAPK-AP1 to reduce *ADAMTS5* expression. miR-30a expression was downregulated in arthritis patients and was negatively correlated with *ADAMTS5* expression. IL-1β suppressed miR-30a expression by recruiting the AP-1 transcription factor c-jun/c-fos to the miR-30a promoter [27]. Therefore, *ADAMTS5* might be regulated by various factors which might be the reason why we cannot observe its reduction.

Nowadays, RA patients have been well treated with biological agents, and it is difficult to collect human RA fibroblasts samples from patients in a clinical setting. To make up for this deficiency, we used the collagen loss by Cf-02 in an arthritis animal model to further explore mechanisms in vivo. In vivo testing using a collagen-II induced edema method revealed that Cf-02 suppresses inflammation and cartilage damage. In our study, aside from TNF-α stimulation the primary chondrocytes from porcine and human, which is the common model to study rheumatoid arthritis disease, the collagen-II induced edema method was also used to verify the beneficial effects of Cf-02. In vivo, Cf-02 was shown to suppress inflammation and cartilage damage (Figure 6). Nevertheless, our results suggest that Cf-02 may have the potential to act as a lead compound in the subsequent identification of novel compounds. Moreover, we anticipate that our study will initiate further in vitro and in vivo research with the aim of confirming the therapeutic benefits of Cf-02 in patients with arthritis and inflammation-mediated joint disorders.

## 4. Materials and Methods

### 4.1. Reagents and Antibodies

TNF-α was supplied by an R & D commercial company (Canandaigua, NY, USA). The polyclonal antisera against iNOS (catalog number: SC-651), MMP-13 (catalog number: SC-30073), and aggrecan neoepitope (catalog number: NB100-74350) antibodies were purchased from Santa Cruz Biotechnology (Santa Cruz, CA, USA) and Novus Biologicals, (Littleton, CO, USA). Hsu-Shan Huang synthesized the small-molecule inhibitor (SMI) and provided the Cf-02 used in this study. The small molecules were reduced to concentrations that were suitable for individual experiments by diluting the stock preparation with culture medium.

### 4.2. Isolation and Culture of Porcine and Human Chondrocytes

Porcine cartilage specimens were taken from the hind leg joints. Chondrocytes were prepared from cartilage according to the methods outlined in a previous report [28]. Briefly, articular cartilage underwent enzymatic digestion using 2 mg/mL protease in serum-free Dulbecco’s modified Eagle’s medium (DMEM)/antibiotics followed by collagenase I (2 mg/mL) and hyaluronidase (0.9 mg/mL) in DMEM with fetal bovine serum (FBS) digestion overnight. Cells were collected via a cell strainer (Beckton Dickinson, Mountain View, CA, USA) and cultured in DMEM that contained 10% FBS and antibiotics for 3–4 days prior to use.

Human chondrocytes were harvested using cartilage from patients with osteoarthritis who underwent total knee replacement aseptically, human chondrocytes samples were obtained following protocols approved by the Institutional Review Board (IRB) of Tri-Service General Hospital, National Defense Medical Center Institutes Human Ethics Committee code: 1-102-05-091; Date: 02/09/2013. Chondrocytes were prepared as previously described. [29]. Briefly, articular cartilage was made into 0.5 cm^2^ pieces. The protease (2 mg/mL) (EMD Millipore, Billerica, MA, USA) was for enzyme digestion at 37 °C with 5% CO_2_ for 1 h, whereupon the specimens underwent digestion overnight using 0.25 mg/mL collagenase I and 500 U/mL hyaluronidase in DMEM medium containing 10% fetal bovine serum. Cells were collected using a cell strainer and seeded at concentrations of 6–8 × 10^6^ cells in T75 flasks within DMEM containing 10% FBS and antibiotics for 3–4 days prior to use.

### 4.3. Cytotoxicity Analysis and Measurement of NO Concentrations

The concentration of released LDH was used as an indicator of damage to the plasma membrane according to the manufacturer’s instructions (Roche, Indianapolis, IN, USA). The percentage of cytotoxicity was calculated as: ((sample value − medium control)/(high control − medium control)) × 100. Single sample values comprised the averages of absorbance values obtained in triplicate from treated culture supernatants following the subtraction of the absorbance values associated with the background control. The average absorbance values of untreated cell culture supernatants (used as control mediums) were calculated in a similar manner. Equal quantities of cells treated with 1% Triton X-100 were adopted as the high control. The amount of NO released was derived from its stable end product (nitrite) in the supernatant [28]. We performed the Griess reaction to determine the concentration of nitrite using a spectrophotometer.

### 4.4. Nuclear Extract Preparation and EMSA

Nuclear extract preparation and EMSA analysis were performed in accordance with methods described in our previous report [29]. Oligonucleotides containing the NF-κB, STAT-3, IRF-1, and AP-1 binding sites were used as a DNA probes. The detailed steps for the EMSA experiment were performed as described in our previous report [30].

### 4.5. Real-Time RT-PCR and Western Blotting

Total RNA was isolated after cells were lysed using Trizol reagent (Invitrogen; Carlsbad, CA, USA) and RNA samples were treated with DNase I (Roch, Indianapolis, IN, USA) prior to reverse transcription in accordance with the manufacturer’s protocol. Total RNA (2 μg) was then reverse transcribed into cDNA using the Superscript First-Strand Synthesis System (Invitrogen, Grand Island, NY, USA). The mRNA gene expression was measured and duplicated thrice by real-time RT-PCR measurements in accordance with the manufacturer’s instructions (power SYBR Green PCR Master Mix, Applied BioSystems, Foster City, CA, USA). The primer sequences for these genes were either designed by us or described by other researchers [31,32]. The primers sequences are listed in supplementary Table S1. The reactions underwent 50 cycles at 95 °C for denaturation and at 60 °C for annealing and extension. For this, the ABI Prism 7000 Sequence Detection system (Applied BioSystems) was used. After the data were collected, we calculated changes in gene expression following stimulation with TNF-α or IL-1 in the presence or absence of Cf-02 using the following formula: fold changes = 2^−ΔΔ*C*t^, where Δ*C*_t_ = *C*_t targeted gene_ − *C*_t *GAPDH*_, and Δ(Δ*C*_t_) = Δ*C*_t stimulated_ − Δ*C*_t control_.

Enhanced chemiluminescence (ECL) Western blotting (Amersham-Pharmacia, Arlington Heights, IL, USA) was performed according to previous study description [29]. The protein was separated by 10% sodium dodecyl sulfate-polyacrylamide gel electrophoresis (SDS-PAGE) and then transferred to a nitrocellulose filter to analyze equal amounts of whole cellular extracts. For immunoblotting, the nitrocellulose filter was incubated in Tris-buffered saline for 1 h, and further blotting with antibodies against specific proteins for 2 h at room temperature. After being washed using milk buffer, the filter was incubated with rabbit anti-goat IgG (1:5000) or goat anti-rabbit IgG (1:5000) conjugated to horseradish peroxidase for 30 min. Finally, the filter was incubated with substrate and exposed to X-ray film (GE Healthcare, Buckinghamshire, UK).

### 4.6. Gelatin Zymography

Gelatin zymography was performed as previously described [22] with some modifications. Specifically, culture supernatant (16 μL) was mixed with (1) 4 μL buffer containing 4% SDS, (2) 0.15 M Tris (pH 6.8), and (3) 20% glycerol containing 0.05% bromophenol blue. A 10% polyacrylamide gel was copolymerized with 0.1% gelatin (Sigma-Aldrich, St. Louis, MO, USA); the supernatant mixture was then analyzed. After electrophoresis, gels were washed with 2.5% Triton X-100 3 times for 20 min. After incubation with the gelatinase buffer for 24 h at 37 °C, the gel was stained with 0.1% Coomassie blue. Under the background of uniform light blue staining clear bands demonstrating genatinolytic activity were found. The localization of proMMP-13 and MMP-13 was evaluated using Alpha EaseFC software (Alpha Innotech Corp, San Leandro, CA, USA) according to standard molecular weights and previous reports by other researchers [33].

### 4.7. Preparation of Cartilage Explants and Analysis of Cartilage Degradation

The preparation of cartilage explants was performed using the methods outlined in our previous report [28]. Briefly, articular cartilage from the femur head of the hind limb joint of pigs was excavated using a stainless steel dermal-punch that measured 3 mm in diameter (Aesculap, Tuttlingen, Germany). Following this, the extracted articular cartilage was weighed. For the dissection, each cartilage explant was cultured in DMEM and contained antibiotics and 10% FBS in a 24-well plate. Cartilage explants were then allowed to rest for 72 h in serum-free DMEM before undergoing further study. The degradation of cartilage was evaluated using a measure of proteoglycan that had been released into the cell culture medium [28]. Briefly, the 1,9-dimethylmethylene blue (DMB) solution (Sigma-Aldrich) was added to the culture medium in which the metachromatic dye was bound with sulfated glycosaminoglycan (GAG), which is a major component of proteoglycan. We then measured the quantity of the GAG-DMB complex that formed in a 96-well plate using a plate reader (TECAN Safire, TECAN Austria GmbH, Grödig, Austria) at a wavelength of 595 nm. Finally, the loss of GAG and total GAG released per mg of cartilage were calculated.

### 4.8. Safranin-O Staining, IHC Staining, and Measurement of Aggrecan NITEGE Neoepitopes

Cartilage explants were placed in embedding medium (Miles Laboratories, Naperville, IL, USA) and rapidly frozen at −80 °C, continuous and discontinuous microscopic sections (7 μm) of cartilage explants were cut at −20 °C and mounted on Superfrost Plus glass slides (Menzel-Gläser, Braunschweig, Germany). These slices were used for evaluation changes in proteoglycan content by Safranin-O/fast green, countered with Weigert’s iron hematoxylin staining [28]. The expression of MMP-13 and aggrecan NITEGE neoepitopes recognized was determined as described using MMP-13 and NITEGE antibodies in tissue slices [28].

### 4.9. Collagen-Induced Arthritis Model

Male SD rats (6–8 weeks) were housed in a 12:12-h light-dark cycle at 22 °C and allowed free access to standard rat chow and water. For the experiment, animals were first randomly divided into 3 groups. All animals then received a subcutaneous injection of 150 μg bovine collagen type II in 200 μL of 0.01 M acetic acid solution and complete Freund’s adjuvant (CFA) (at a ratio of 1:1) at the base of the tail. On day 7, the rats received a booster injection of 150 μg covine collagen type II in 100 uL of 0.01 M acetic acid solution and incomplete Freund’s adjuvant (CFA) at a ratio of 1:1. Clinical signs of footpad swelling and arthritic scores were monitored for 24 days. On starting, 7 days before collage II injection, and on days 1–22, rats were intraperitoneal injection treated with a dosage of Cf-02 (10 mg/kg) and Quercetin (20 mg/kg) dissolved in poly (ethylene glycol) 400. The experiment protocol was approved by the DCB institutional animal care and use committee (IACUC). Key equipment included a disperser (T 10 basic ULTRA-TURRAX^®^) (Sigma-Aldrich), digimatic caliper (Series No.500, Mitutoyo Corp., Tokyo, Japan), and body weight scale. The body weight of the animals was determined twice a week for three weeks. Paw thickness was measured using a caliper twice a week for three weeks. Arthritic scores ranged from 0 to 5; scoring was carried out as previously described [34].

### 4.10. Statistical Analysis

Wherever necessary, results were expressed as mean ± SD. Unpaired Student’s *t*-tests were used to identify statistically significant differences, where *p* < 0.05 was considered significant.

## Supplementary Materials

The following are available online at http://www.mdpi.com/1422-0067/19/5/1453/s1.

## Abbreviations

TNF-α	tumor necrosis factor-alpha
HS-Cf	2-hydroxy-*N*-[3-(trifluoromethyl)phenyl]benzamide
Cf-02	6-(2,4-difluorophenyl)-3-(3-(trifluoromethyl)phenyl)-2*H*-benzo[*e*][1,3]oxazine-2,4(3*H*)-dione
iNOS	nitric oxide synthase
NO	nitric oxide
MMPs	matrix metalloproteinases
ADAMTS	aggrecanases like a disintegrin and metalloproteinase with thrombospondin motifs
IRF-1	interferon regulatory factor-1
NF-κB	nuclear factor-kappaB
AP-1	activator protein-1
STAT-3	signal transducer and activator of transcription-3
