# Supplementary material for: Arthroprotective Effects of Cf-02 Sharing Structural Similarity with Quercetin
Source: Int J Mol Sci. 2018 May 14;19(5):1453. doi: 10.3390/ijms19051453 (PMC5983747; doi:10.3390/ijms19051453)
Supplement: Supplementary file 1 [file ijms-19-01453-s001.docx]

Supplementary Materials: Arthroprotective Effects of Cf-02 Sharing Structural Similarity with Quercetin

**Table S1.** Sequences of oligonucleotides that were used in the present study.

| **Gene Name** | **Primers** | **Sequence (5’–3’)** |
| --- | --- | --- |
| *MMP-1* | Forward | AAGCAGACATAATGATATCCTTTGTCA |
|  | Reverse | TGAGCATCCCCTCCAATACCT |
| *MMP-3* | Forward | AGAAGTTCCTGGGGTTGGAGG |
|  | Reverse | TCTTGGAGAATGTAAGCGGAG |
| *MMP-13* | Forward | GATCCCCATTTTGATGATGATGAA |
|  | Reverse | GTCTTCATCTCCTGGACCATAGAGAGA |
| *ADAMTS4* | Forward | CAGGGTCCCATGTGCAACGT |
|  | Reverse | CATCTGCCACCACCAGGGTCT |
| *ADAMTS5* | Forward | TTCGACATCAAGCCATGGCAACTG |
|  | Reverse | AAGATTTACCATTAGCCGGGCGG |
| *TIMP-2* | Forward | GTAGTGATCAGGGCCAAAGC |
|  | Reverse | TTCTCTGTGACCCAGTCC |
| *GAPDH* | Forward | GTCATCCATGACAACTTCGG |
|  | Reverse | GCCACAGTTTCCCAGAGG |
